# Supplementary material for: Evaluation of Immunomodulatory Responses and Changed Wound Healing in Type 2 Diabetes—A Study Exploiting Dermal Fibroblasts from Diabetic and Non-Diabetic Human Donors
Source: Cells. 2021 Oct 28;10(11):2931. doi: 10.3390/cells10112931 (PMC8616411; doi:10.3390/cells10112931)
Supplement: Supplementary file 1 [file cells-10-02931-s001.zip › cells-1387491-supplementary.pdf]

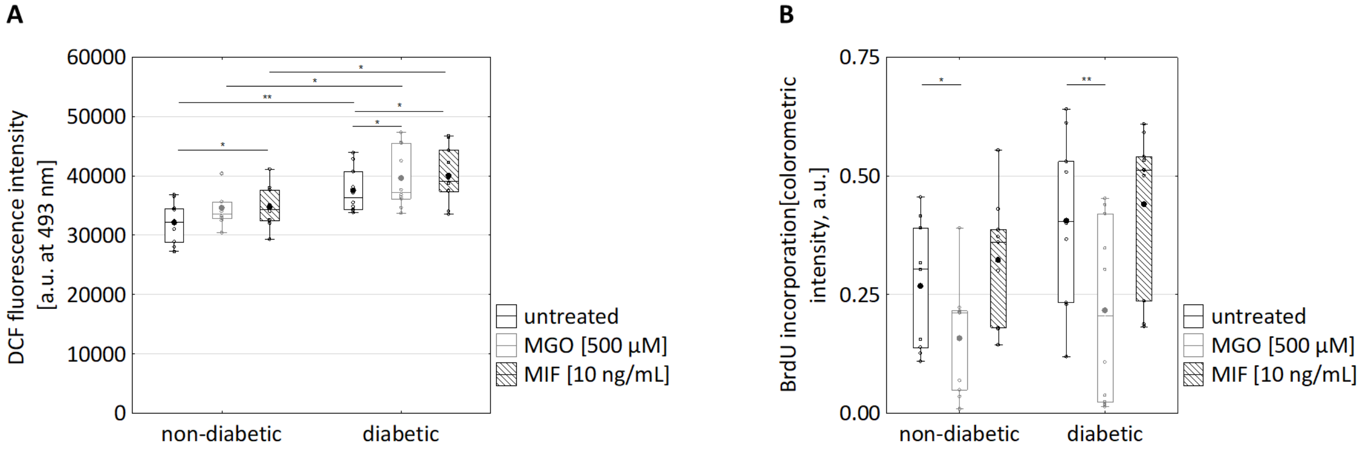

Figure S1. Effects of MGO and MIF treatment on the ROS formation and proliferation of dermal fibroblasts Cultured fibroblasts from diabetic and non-diabetic donors were treated with 500 µM MGO (diagonal stripes) or 10 ng/mL MIF (grey) or left untreated (white) for 24 h. A) The formation reactive oxygen species (ROS) was detected in terms of DCF. B) Proliferation was analyzed in terms of BrdU incorporation $*p < 0.05$, $**p < 0.01$ Statistical significance was determined by Mann-Whitney-U-Test between groups and by Wilcoxon for comparison between untreated and treated. $n_{non\text{-}diabetic}$=10; $n_{diabetic}$=10.

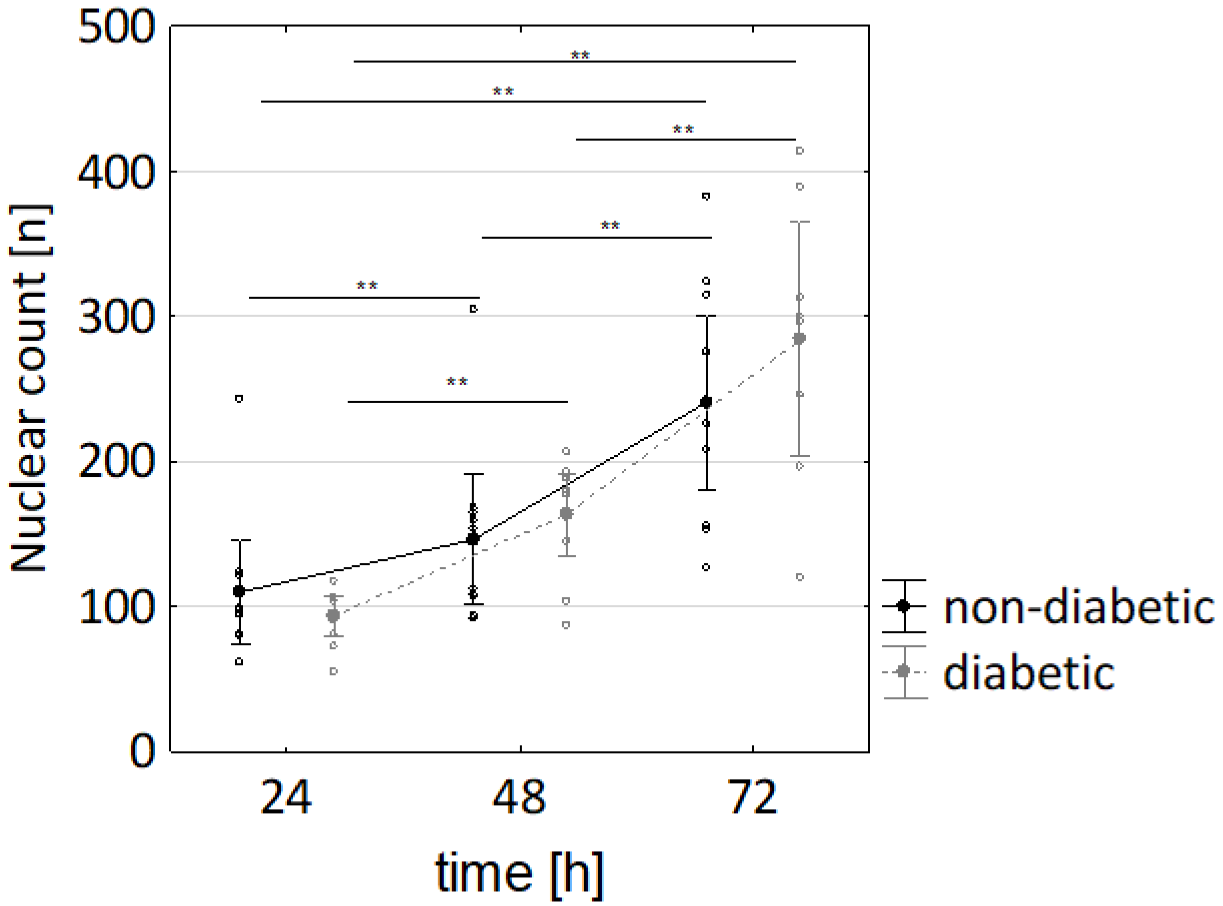

Figure S2. Comparable doubling rates of dermal fibroblasts from diabetic and non-diabetic donors. Cultured fibroblasts from diabetic and non-diabetic donors were stained with Hoechst 33342 after 24h, 48h and 72h. The number of nuclei was determined by manual counting. . $n_{non\text{-}diabetic}$=10; $n_{diabetic}$=10.

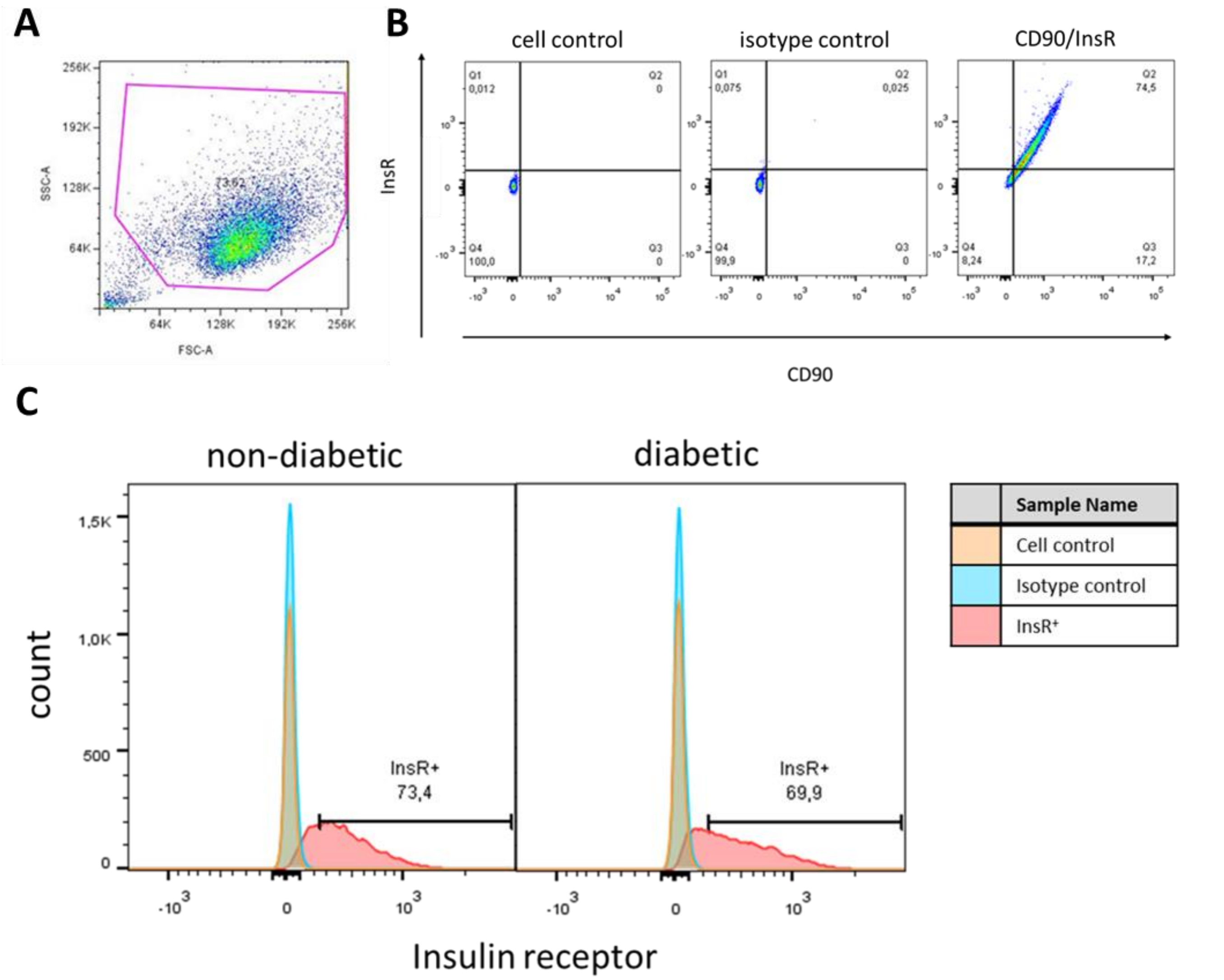

Figure S3. FACS analysis of surface insulin receptor staining. A Representative scatter plot of dermal fibroblasts, **B** representative dot plots of cell control, isotype control and co-staining for CD90 as a marker for fibroblasts and insulin receptor (InsR), **C** representative histograms showing InsR expression in $CD90^+$ fibroblasts. $n_{non\text{-}diabetic}$=8; $n_{diabetic}$=10.

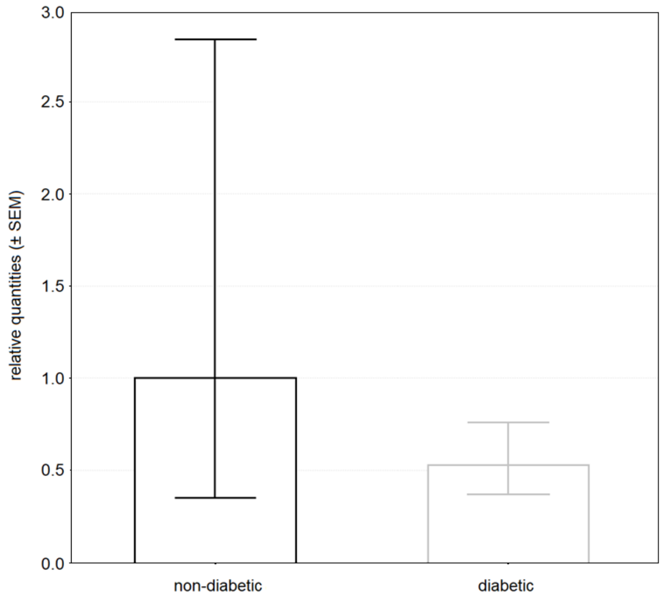

Figure S4. Increased gene expression of procollagen C-endopeptidase enhancer 2 (pcolce2). Dermal fibroblasts from diabetic and non-diabetic donors were lysed, RNA was isolated and gene expression was analysed**.** Statistical significance was determined by Mann-Whitney-U-Test between groups. $n_{non\text{-}diabetic}$=4; $n_{diabetic}$=10 .
